# Role of Computed Tomography Angiography in the Short-Term Follow-up of Aortic Coarctation Repair

**DOI:** 10.21470/1678-9741-2023-0220

**Published:** 2024-01-30

**Authors:** Mariana Ribeiro Rodero Cardoso, Ariela Maltarolo Crestani, Antônio Soares Souza, Fernanda Del Campo Braojos Braga, Marília Maroneze Brun, Alexandre Noboru Murakami, Francisco Candido Monteiro Cajueiro, Carlos Henrique De Marchi, Ulisses Alexandre Croti

**Affiliations:** 1 Radiology Service, Hospital da Criança e Maternidade (HCM), Fundação Faculdade Regional de Medicina de São José do Rio Preto (FUNFARME), Faculdade de Medicina de São José do Rio liPreto (FAMERP), São José do Rio Preto, São Paulo, Brazil; 2 CardioPedBrasil® - Centro do Coração da Criança, Hospital da Criança e Maternidade (HCM), Fundação Faculdade Regional de Medicina de São José do Rio Preto (FUNFARME), Faculdade de Medicina de São José do Rio Preto (FAMERP), São José do Rio Preto, São Paulo, Brazil; 3 Cardiology Surgery Department, Serviço de Cirurgia Cardíaca do Norte do Paraná, Universidade Estadual de Londrina (UEL), Londrina, Paraná, Brazil

**Keywords:** Aortic Coarctation, Recoarctation, Computed Angiotomography, Congenital Heart Defect

## Abstract

**Introduction:**

Coarctation of the aorta (CoA) is a narrowing of the thoracic aorta that often manifests as discrete stenosis but may be tortuous or in long segment. The study aimed to evaluate pre and post-surgical aspects of pediatric patients submitted to CoA surgical correction and to identify possible predisposing factors for aortic recoarctation.

**Methods:**

Twenty-five patients were divided into groups according to presence (N=8) or absence (N=17) of recoarctation after surgical correction of CoA and evaluated according to clinical-demographic profile, vascular characteristics via computed angiotomography (CAT), and other pathological conditions.

**Results:**

Majority of males (64%), ≥ 15 days old (76%), ≥ 2.5 kg (80%). There was similarity between groups with and without recoarctation regarding sex (male: 87% vs. 53%; P=0.277), age (≥ 15 days: 62.5 vs. 82%; P=0.505), and weight (≥ 2.5 kg: 87.5 vs. 76.5; P=0,492). Altered values of aortic root/Valsalva diameter, proximal transverse arch, and distal isthmus, and normal values for aorta prevailed in preoperative CAT. Normal values for the aortic root/Valsalva sinus diameter were observed with and without recoarctation, the same for both groups regarding ascending and descending aorta in postoperative CAT. No significant difference for altered values of proximal transverse arch and alteration in distal isthmus was observed.

**Conclusion:**

No predictive risk for recoarctation was observed. CTA proved to be important in CoA diagnosis and management, since CoA is mainly related with altered diameter of aortic root/sinus of Valsalva and proximal and distal aortic arch/isthmus, however, it failed to show predictive risk for recoarctation.

## INTRODUCTION

**Table:** 

Abbreviations, Acronyms & Symbols	
CAT	= Computed angiotomography
CI	= Confidence interval
CoA	= Coarctation of the aorta
FAMERP	= Faculdade de Medicina de São José do Rio Preto
FUNFARME	= Fundação Faculdade Regional de Medicina de São José do Rio Preto
HCM	= Hospital da Criança e Maternidade
PDA	= Patent ductus arteriosus
SD	= Standard deviation

Coarctation of the aorta (CoA) is a congenital heart defect defined as a narrowing of the thoracic aorta that often manifests as a discrete stenosis but may be presented as tortuous or in a long segment. It is typically located distal to the left subclavian artery but it can also be located distant to the ductus^[1,2]^.

CoA is the fifth most common congenital cardiopathy, with an estimated prevalence of one in 2,500 live births and a 2:1 predominance in males^[3]^. This vessel malformation can cause premature death if maintained without correction, with 50% non-treatment mortality at 30 years, 75% at 46 years, and 90% at 58 years of age^[4]^.

Surgical correction has been the procedure of choice for CoA neonatal treatment and is indicated at the time CoA is diagnosed^[5]^. Several advances have been achieved in past decades, such as low overall mortality rate in spite of moderate morbidity observed within the first 30 postoperative days, especially in patients younger than one year old^[6]^. However, the outcomes of CoA surgical correction are not always benign^[2]^.

Aortic recoarctation is one of the common post-surgical morbidities in patients submitted to CoA surgical correction, with incidence up to 34% in the adult population^[7,8]^. The recurrence of coarctation after repair is also observed in children and is commonly assigned with inadequate growth of the aorta wall at the site of repair since the surgery might be performed before the aorta reaches its mature size^[2,9]^.

Though transthoracic echocardiography is the first choice of imaging exam, this technique has some limitations when bone deformities are present, and also when the analysis of extracardiac and collateral circulation structures is necessary^[10,11]^. And although the accuracy of coarctation diagnosis has improved, it still remains challenging in the prenatal period^[12]^. In this sense, computed angiotomography (CAT) offers several advantages, such as optimal spatial resolution and fast acquisition of images, that might improve the assertiveness of the diagnosis^[13,14]^.

This study aimed to investigate anatomical aspects of the aorta by CAT in two moments: (i) prior to CoA surgical correction; and (ii) post-surgery, within one year period, investigating the possible association between anatomical aspects of the thoracic aorta, bone deformities, genetic alterations, and other cardiovascular alterations to define predictive risk factors for aortic recoarctation in pediatric patients submitted to the surgical correction of CoA.

## METHODS

### Ethical Aspects

The study was approved by the Ethics Committee of Faculdade de Medicina de São José do Rio Preto (CEP/FAMERP - 91442218.80000.5415) and a Free and Informed Consent Term was obtained.

### Patients

We reviewed the local cardiology registry to identify all children diagnosed with CoA between January 2005 and December 2018 at the CardioPedBrasil®, Centro do Coração da Criança, Hospital da Criança e Maternidade (HCM), Fundação Faculdade Regional de Medicina de São José do Rio Preto/Faculdade de Medicina de São José do Rio Preto (FUNFARME/FAMERP). We then searched for the radiology picture archiving and communication system (Agfa Impax 6^TM^) to identify all imaging studies performed before the surgery and within one year after the surgical correction.

### Inclusion Criteria

The criteria for patient inclusion were: (i) age between 0 and 18 years (pediatric population), (ii) have received medical assistance in our health unit, (iii) have a diagnosis of CoA, (iv) have received surgical correction for the CoA condition, and (v) have performed a preoperative and a postoperative CAT (within one year after CoA’s surgical correction).

In this period, 170 patients diagnosed with congenital CoA were operated by a single cardiac surgeon at our service. From 170 patients, 25 (14.7%) were submitted to CAT based on their echocardiogram, that showed significant alterations, such as a peak in the pressure gradient > 20 mmHg suggesting possible aortic recoarctation.

### Clinical-Demographic and Anatomic Characteristics

Clinical-demographic information such as gender, age, weight, presence of other cardiovascular anomalies, bone deformities, and genetic syndromes were analyzed from the patients’ electronic medical records.

We considered the vascular anatomical characteristics of the thoracic aorta including the root of the sinus of Valsalva, ascending aorta, crotch at the levels of the proximal transverse arch, distal isthmus, and descending portion. Data were collected prior to the surgery for CoA’s correction (preoperative CAT) and after CoA’s correction surgery (postoperative CAT), when the echocardiogram showed indicatives of possible recoarctation. Postoperative CAT were performed within a one-year period after the surgery for CoA correction.

The cardiovascular anomalies considered in this study were: bicuspid aortic valve, subvalvar or supravalvar aortic stenosis, atrioventricular septal defect, tricuspid atresia, mitral hypoplasia, hypoplastic left heart syndrome, Shone syndrome, *cor triatriatum*, right ventricular outflow tract, interatrial communication, interventricular communication, aberrant right subclavian artery, vascular variants of supra-aortic branches, persistent left superior vena cava, anomalous pulmonary vein connections, and heterotaxy syndromes.

### Thoracic Aorta Computed Angiotomography Study

Thoracic aorta CAT study was performed at the Radiology Service of the HCM/FUNFARME/FAMERP, using a 64-channel device (Toshiba Medical System Corporation - Aquilion Model TSX-101A) with image acquisition immediately after intravascular infusion of non-ionic iodinated contrast medium water solution (Iobitridol 350 mgI/mL). Axial sections without angulation were made from the low cervical region to the height of the adrenal glands.

**Fig. 1:**
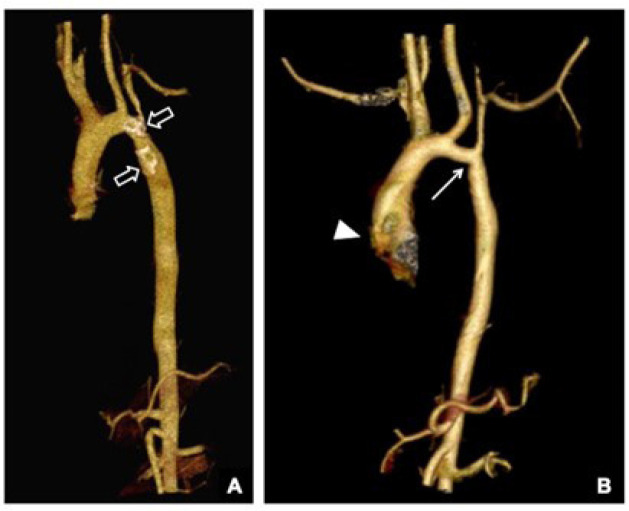
Computed angiotomography images of the thoracic aorta (three-dimensional reconstructions) of patients previously submitted to surgical correction of coarctation. (A) Exam of a six-year-old male patient performed one year after the surgical treatment, showing a satisfactory anatomical aspect of the thoracic aorta that has a preserved caliber throughout its extension, noting postoperative parietal calcifications in the distal isthmus, and in the descending aorta (empty arrows). (B) Exam a four-year-old male patient, one year after the operation, showing segmental recoarctation of the aortic arch, with greater narrowing point at the level of the distal isthmus (long white arrow), and retrograde dilatation of the sinus of Valsalva (arrowhead).

Parameters were adjusted as cutting thickness, 3 mm; increment, 3 mm; pitch (distance traveled by the examination table during a 360° rotation of the x-ray tube), 0.6 to 1.5 mm; field of view (or FOV) appropriate to the region of interest; KV (kilovolt), 120; and mAs (milliamperes) with automatic dose modulation, the lowest possible necessary for the required image quality, according to the ALARA (or As Low As Reasonably Achievable) principle of radiation dose optimization^[15]^. Post-processing of the images obtained was performed to generate a three-dimensional reconstruction of the aorta, facilitating the comprehension of the surgical anastomosis’ region, possible recoarctation, and post-stenotic dilatation of the aorta (Figure 1).

CAT imaging was interpreted by a single radiologist with specialization in pediatric radiology and subspecialty in congenital cardiac imaging. We performed multiple measurements of the thoracic aorta using double-oblique multiplanar reconstructions of the images obtained, allowing the definition of the true axial axis of the vessel for a reliable diameter measurement. The following portions of the thoracic aorta were selected for measurement: aortic root at the level of the sinus of Valsalva, ascending aorta, aortic arch at the level of the proximal transverse arch, distal isthmus, and descending aorta at the proximal and distal thirds.

The measurements were indexed by body surface area for each patient, with normal values considered within the standard deviation (*z*-score) between -2 and +2. Data were used to characterize the selected patients and to compare the group of children with (after CoA surgical repair) and without recoarctacion.

### Statistical Analysis

Descriptive analysis of variables was presented as percentage and frequency values. Qualitative variables were analyzed using Fisher’s exact test or Chi-square test (χ2). Mean values were compared by *t*-test or Mann-Whitney U test. Logistic regression evaluated the chance of the event (recoarctation) occurrence in the presence of different variables, using a multiple comparison test. An α error of 5% was admitted with a *P*-value < 0.05 as significant.

## RESULTS

Sex, age, and weight data of CoA patients with or without recoarctation are presented in Table 1. There was a higher frequency of male individuals (64%), with 15 days of age or older (76%), and weighing ≥ 2.5 kg (80%) between CoA patients independent of the recoarctation status. The mean lifetime of all patients was 18 ± 1730 days, with similar values when comparing those with (20 ± 30 days) and without (78 ± 1977 days; *P*=0.500) recoarctation. Mean weight of the children was 4.1 ± 15.2 kg, with no difference between patients with (3.27 ± 1.94 kg) and without (3.89 ± 17.78 kg; *P*=0.476) recoarctation.

**Table 1:** Clinical-demographic profile of patients with aortic coarctation with or without recoarctation.

Characteristics	Total (N=25)		Recoarctation				*P*-value
			Yes (N=8)		No (N=17)		
	N	%	N	%	N	%	
Sex							0.277
Male	16	64	7	87.5	9	53	
Female	9	36	1	12.5	8	47	
Age (days)							
Mean ± SD	18 ± 1730		20 ± 30		78 ± 1977		0,5
< 15	6	24	3	37.5	3	18	0.505
≥ 15	19	76	5	62.5	14	82	
Weight (kg)							
Mean ± SD	10.4 ± 14.5		3.2 ± 1.94		3.8 ± 15.7		0.476
< 2.5	5	20	1	12.5	4	23.5	0.492
≥ 2.5	20	80	7	87.5	13	76.5	

SD=standard deviation

There was similarity between the groups with and without recoarctation, respectively, for frequencies of sex (male: 87.5% and 53%), age (≥ 15 days: 62.5% and 82%), and weight (≥ 2.5 kg: 87, 5% and 76.5%; *P*>0.05, for all comparisons).

**Fig. 2:**
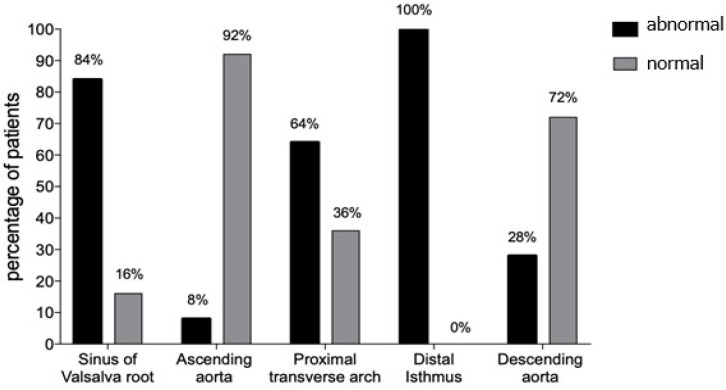
Distribution of patients with coarctation of the aorta according to vascular characteristics assessed by preoperative computed tomography angiography. Abnormal means altered diameter, and normal means normal diameter.

The anatomical characteristics of the preoperative CAT study is shown in Figure 2. Altered values for root diameter at the level of the sinus of Valsalva and proximal and distal isthmus prevailed in the studied population (84%, 64%, and 100%, respectively). Moreover, normal values stood out for the ascending (92%) and descending aorta diameter (72%).

Anatomical characteristics of the postoperative CAT imaging, considering patients with (N=8; 32%) and without (N=17; 68%) recoarctation are shown in Table 2.

**Table 2:** Distribution of patients with aortic coarctation according to vascular characteristics assessed by post-surgery computed tomography angiography considering the groups with and without recoarctation.

Vascular characteristic	Recoarctation				*P*-value
	Yes (N=8)		No (N=17)		
	N	%^*^	N	%^*^	
Sinus of Valsalva root					0.283
Altered	3	37.5	2	13.3	
Normal	5	62.5	15	86.7	
Ascending aorta					1.000
Altered	1	12.5	1	6.3	
Normal	7	87.5	16	93.7	
Proximal transverse arch					0.080
Altered	2	25	12	70.5	
Normal	6	75	5	29.5	
Distal isthmus					0.268
Altered	8	100	13	76.4	
Normal	0	0	4	23.6	
Descending aorta					0.639
Altered	3	37.5	4	23.5	
Normal	5	62.5	13	76.5	

* Percentage of patients based on the total sample of the corresponding group

Both groups, with or without recoarctation, showed normal values for aortic root at the level of the sinus of Valsalva (62.5% of patients with recoarctation and 86.7% of patients without recoarctation; *P*=0.283). The same occurred for the ascending aorta (87.5% of patients with recoactation and 93.7% of patients without recoarctation; *P*=1.00) and for the descending aorta (62.5% of patients with recoactation and 76.5% of patients without recoarctation; *P*=0.639).

Regarding the aortic arch, altered values of the proximal isthmus were observed mainly in the group without recoarctation (70.5%), compared to those with recoarctation (25%), although without significant difference (*P*=0.08), whereas alterations in the distal isthmus prevailed in both groups (100% and 76.4%, respectively; *P*=0.268).

The distribution of associated diseases of CoA patients with and without recoarctation are shown in Table 3. Other cardiovascular anomalies prevailed in patients without recoarctation (82.4%), compared to those with recoarctation (12.5%; *P*=0.001). Bone deformities were not detected, and genetic syndromes were observed in only three patients without recoarctation (17.6%; *P*=0.527).

**Table 3:** Pathological conditions in patients with coarctation of the aorta and in groups with and without recoarctation.

Characteristics			Recoarctation				*P*-value
	Total (N=25)		Yes (N=8)		No (N=17)		
	N	%	N	%	N	%	
Other cardiovascular anomalies^*^							0.001
Yes	15	60	1	12.5	14	82.4	
No	10	40	7	87.5	3	17.6	
PDA							0.641
Yes	20	80	6	75	14	82.4	
No	5	20	2	25	3	17.6	
Bone deformities							N/A
Yes	0	0	0	0	0	0	
No	25	100	8	100	17	100	
Genetic syndromes^**^							0.527
Yes	3	12	0	0	3	17.6	
No	22	88	8	100	14	82.4	

N/A=not applicable; PDA=patent ductus arteriosus

* Other cardiovascular anomalies: bicuspid aortic valve, subvalvar or supravalvar aortic stenosis, atrioventricular septal defect, tricuspid atresia, mitral hypoplasia, hypoplastic left heart syndrome, Shone syndrome, *cor triatriatum*, right ventricular outflow tract, interatrial communication, interventricular communication, aberrant right subclavian artery, vascular variants of supra-aortic branches, persistent left superior vena cava, anomalous pulmonary vein connections, and heterotaxy syndromes

** Turner syndrome, Down syndrome, and Williams syndrome

The logistic regression analysis based on the pathological conditions (such as cardiovascular anomalies, patent ductus arteriosus, and genetic syndromes) did not find significant difference in the identification of independent factors for recoarctation (*P*>0.05; Table 4).

**Table 4:** Logistic regression analysis considering other cardiovascular anomalies, patent ductus arteriosus, and genetic syndromes in patients with aortic coarctation with and without recoarctation assessed by computed tomography angiography.

Characteristic	Odds ratio	95% CI	*P*-value
Other cardiovascular abnormalities^*^	0.000018	11.4-32 - 27.822	0.732
PDA	7,643.8	49.3-24 - 11.831	0.779
Genetic syndromes^**^	5.7	26.5-32 - 37.326	0.866

CI=confidence interval; PDA=patent ductus arteriosus

* Other cardiovascular anomalies: bicuspid aortic valve, subvalvar or supravalvar aortic stenosis, atrioventricular septal defect, tricuspid atresia, mitral hypoplasia, hypoplastic left heart syndrome, Shone syndrome, *cor triatriatum*, right ventricular outflow tract, interatrial communication, interventricular communication, aberrant right subclavian artery, vascular variants of supra-aortic branches, persistent left superior vena cava, anomalous pulmonary vein connections, and heterotaxy syndromes

** Turner syndrome, Down syndrome, and Williams syndrome

CoA diameter, defined as the narrowest aortic diameter observed in the preoperative CAT study, was compared between patients who developed and those who did not develop recoarctation after surgical repair.

**Fig. 3:**
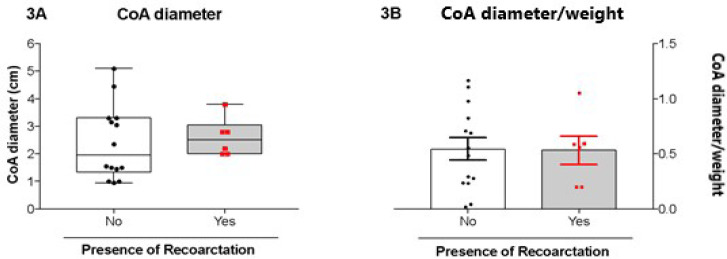
Distribution of the diameter of the coarctation segment of patients operated for coarctation repair who did or did not develop recoarctation after the surgical intervention (3A). Mean and distribution of the diameter of the coarctation segment indexed to the body weight of patients who did or did not develop recoarctation after the surgical intervention (3B). Diameter was measured in cm. CoA=coarctation of the aorta.

The median diameter was 1.95 in the group that did not develop recoarctation, whereas for the patients that suffered recoarctation, it was observed a median of 2.5. No significant difference was observed between the groups (*P*=0.586; Figure 3A).

The diameter of the coarctation segment indexed to the patient’s body weight (CoA diameter/weight) was also compared between the aforementioned groups. The mean CoA diameter/weight observed for the group without recoarctation was 0.545 ± 0.102, and for the group that suffered recoarctation, it was 0.532 ± 0.129. There was no significant difference between groups (*P*>0.1; Figure 3B).

## DISCUSSION

The diagnosis of CoA prevailed in male patients in agreement with other studies^[3,4]^. The surgery was performed mostly in patients with 15 days of age or older and weighing ≥ 2.5 kg. These parameters were established as cutoff values, considering the longer survival rate of individuals with these characteristics who underwent surgical correction^[16]^. Thus, the diagnosis of CoA in neonates older than 15 days of age can provide later surgical treatment, as well as sufficient time for body mass gain, as observed.

Our results showed that altered diameter of the root/sinus of Valsalva and proximal and distal isthmus prevailed in the preoperative CAT, in agreement with other study^[17]^. A meta-analysis study also showed that pediatric patients with altered diameters (proximal transverse arch and distal isthmus) before surgical intervention present an increased risk of recoarctation^[17]^.

There was a similarity between the calibers of the aortic root at the level of the sinus of Valsalva and of the thoracic aorta in the ascending and descending portions in patients with and without recoarctation. Admittedly, the recurrence of focal stenosis contributes to the retrograde vascular dilation of the aorta prior to recoarctation and courses with concomitant post-stenotic dilation^[18]^.

After surgical correction, normal values for the caliber of the sinus of Valsalva stood out both in patients with and without recoarctation, the same occurred for both groups in relation to the ascending and descending aorta. This profile represents a successful surgical repair in these cases, without the occurrence of remodeling or parietal lesion of the aorta early or within one year after the procedure. Importantly, the propensity for dilation and even dissection of the ascending and descending aorta in patients with congenital CoA has been recognized^[18]^, with aortic rupture being the most common cause of death in patients before the current surgical era^[19]^. Also, none of the CoA patients in this study was diagnosed with Marfan syndrome.

The proximal transverse arch presented altered values in both groups with and without recoarctation, yet with no significant difference. Mery et al.^[20]^ followed 290 patients with CoA operated for at least six months and observed that in newborns and infants, the presence of hypoplasia of the proximal transverse arch did not represent a risk factor for the development of hypertension. Thus, it is possible that patients with vascular alteration studied here may not depend on a new surgical intervention, due to the gradual morphological and volumetric development of the thoracic aorta along with the child’s growth.

Alterations in the measurements of the distal isthmus prevailed in the groups with and without recoarctation. However, it was expected that all patients in the recoarctation group would present alterations in the distal isthmus, as it is a *sine qua non* defining condition of the “coarctation” disease^[21]^.

Importantly, many CoA patients without recoarctation although presented alterations in the distal isthmus’ caliber detected by CAT, did not presented hemodynamic repercussions characterized by a pathological increase in systemic blood pressure in the upper limbs, or a significant increase in peak gradient values at transthoracic echocardiogram in the region of restenosis, which would be indicative of a new surgical intervention or even aortic dilation^[22]^. Thus, such alteration may be considered as a residual narrowing of the isthmus, and the patients continued under clinical follow-up by the pediatric cardiology team.

The existence of other cardiovascular abnormalities admittedly increases the mortality rate by up to 20%^[23]^. The presence of such abnormalities may indicate a more aggressive surgical approach through median thoracotomy with the use of extracorporeal circulation, allowing a wide approach to the aorta. However, our data suggest that those cardiovascular abnormalities may not directly influence recoarctation status, considering that only one patient in this group presented other cardiovascular anomalies, compared to the patients without recoarctation.

Considering that the thoracic aorta originates from the 6^th^ embryonic arch, genetic and embryological alterations that occur from the 3^rd^ to the 8^th^ week of gestational period may influence the proper morphological development of this main artery of the body. Also, it allows the development of other vascular variations and associated congenital heart defects^[13,24]^.

Bone deformities were absent in both groups, which would indicate a change in technique or surgical approach. Also, only 12% of CoA patients presented genetic syndromes (Williams, Down, and Turner syndromes), with similar distribution between the groups with and without recoarctation.

Yu et al.^[25]^ recently developed a machine learning model to evaluate the severity of CoA in infants based on anatomical features measured on CTA. Based on the retrospective analysis of CTA and echocardiography of 239 patients, the authors found that the CoA diameter indexed to the body weight was associated with the increased risk of recoarctation, with a hazard ratio of 10.29. Based on this study, we compared the CoA diameter and the CoA diameter indexed to the body weight between patients that developed recoarctation and those who did not develop it. However, our results did not corroborate the findings of Yu et al., since no difference was observed in the comparison between the groups. This divergence may have occurred due to the small sample size of our study compared to the original study^[25]^.

Future studies comparing larger groups may contribute to the advancement of this knowledge and further comprehension of this association between CoA diameter/body weight and risk of recoarctation.

### Limitations

This study has a limitation regarding the sample size. However, it is worth mentioning that one of our objectives was to evaluate the population studied in a pediatric tertiary care hospital in a given period and provide these data in Brazilian population.

## CONCLUSION

The use of CAT study in our population showed that congenital CoA is mainly related with altered diameter of the aortic root/sinus of Valsalva and of the proximal and particularly distal aortic arch/isthmus, proving to be an important exam in the diagnosis and management of this disease. However, CAT did not show any predictive risk for recoarctation in our studied population.

**Table:** 

Authors’ Roles & Responsibilities	
MRRC	Substantial contributions to the conception of the work; and the acquisition, analysis, acquisition, and interpretation of data for the work; drafting the work and revising it; final approval of the version to be published
AMC	Drafting the work and revising it; final approval of the version to be published
ASS	Substantial contributions to the conception and design of the work; revising the work; final approval of the version to be published
FDCBB	Revising the work; final approval of the version to be published
MMB	Substantial contributions to the analysis of data for the work; manuscript revising the work; final approval of the version to be published
ANM	Substantial contributions to the design of the work; and the acquisition, analysis, and interpretation of data for the work; revising the work; final approval of the version to be published
FCMC	Substantial contributions to the acquisition and analysis of data for the work; revising the work; final approval of the version to be published
CHM	Substantial contributions to the acquisition and analysis of data for the work; final approval of the version to be published
UAC	Substantial contributions to the conception and design of the work; revising the work; final approval of the version to be published
